# Parsimonious and efficient assessment of health-related quality of life in osteoarthritis research: validation of the Assessment of Quality of Life (AQoL) instrument

**DOI:** 10.1186/1477-7525-4-19

**Published:** 2006-03-23

**Authors:** Kathryn Whitfield, Rachelle Buchbinder, Leonie Segal, Richard H Osborne

**Affiliations:** 1 Centre for Rheumatic Diseases, Department of Medicine, University of Melbourne, Royal Melbourne Hospital, Parkville, Melbourne, Victoria, Australia; 2 Department of Clinical Epidemiology, Cabrini Hospital and Department of Epidemiology and Preventive Medicine, Monash University, Melbourne, Victoria, Australia; 3 Centre for Health Economics Monash, Faculty of Business and Economics, Monash University, Clayton, Victoria, Australia

## Abstract

**Background:**

The Assessment of Quality of Life (AQoL) utility instrument was psychometrically developed for the general population. This study aimed to explore its potential as an osteoarthritis (OA) outcome measure.

**Methods:**

WOMAC, Lequesne index, SF-36, Visual analogue scales and the AQoL were administered to 222 people with OA. The ability of each questionnaire to detect differences between groups was based on (i) self-rated health (SRH) and, (ii) differences between people on an orthopedic waiting list (WL) vs people with OA in the community (C). Comparisons included effect size, relative efficiency and receiver operator characteristic curves.

**Results:**

All instruments detected differences between groups; however no one instrument exhibited superior efficiency. The AQoL demonstrated strong psychometric properties.

**Conclusion:**

The AQoL has equivalent performance to comparator questionnaires commonly used in OA research and would be a useful adjunct to well-established disease specific scales. The AQoL has important advantages; brevity (12 items), facilitates comparisons between disease groups, and delivers a utility score that can be used in health economic evaluations.

## Background

Osteoarthritis (OA) represents a significant public health problem and disease burden globally, resulting in major disability and pain in affected individuals and significant health care costs for associated disease management [1]. Given the high disability and poor quality of life associated with OA, patient-perceived outcomes are considered important when evaluating interventions. Disease specific outcome measures are favored as they capture specific symptomatology, e.g., pain and stiffness, and functional ability. This is particularly the case for clinical trials [2]. One commonly applied disease-specific scale is the Western Ontario and McMaster Arthritis Index (WOMAC) which provides scores with wide currency, providing researchers with readily interpretable information on OA outcomes [3,4].

In the face of the demands on limited health budgets there is an increasing imperative for cost effectiveness analyses of new technologies. This means outcome measures are required that can establish both the effectiveness of new technologies or new approaches to care and are also suitable as the denominator in cost-effectiveness analyses. This interest extends to comparisons of performance (including impacts on quality of life and mortality) of interventions using cost effectiveness or cost utility analyses across disease categories and disease stages. Disease specific health status outcome measures are unsuitable for these types of analyses. Generic health related quality of life (HRQoL) measures can, in some circumstances, assist with inter-disease group comparisons [5] but cost-utility analyses require generic utility HRQoL instruments [1]. The Assessment of Quality of Life (AQoL) instrument is a generic utility HRQoL tool, developed using stringent psychometric methods and is sensitive to a wide range of health states [6] and has been validated for use in a range of patient groups [7-9]. The AQoL has been demonstrated to have excellent responsiveness in prior studies. In the frail elderly and in people who have had a stroke the AQoL has been shown to be more responsive than the SF-36 and more responsive than stroke-specific instruments [10,11]. This is remarkable given that the AQoL is a short multi-attribute utility instrument.

In this study we assessed the measurement properties of the AQoL in a large sample of people with OA, a common chronic condition, and compared its performance with other commonly used disease specific and a generic HRQoL instruments.

## Methods

The data used in this study were collected as part of a survey of health status of people with OA to assist in priority setting for the disease, conducted in Melbourne, Australia [12]. The sampling frame was designed to reflect the possible range of disease severity of participants in published trials, from severe to mild disease. Participants with clinically diagnosed OA were recruited from a hospital-based orthopedic surgery waiting list, public hospital and private rheumatology (ambulatory care) clinics. A community-based sample of people with self-reported OA were also recruited through membership of the Victorian Arthritis Foundation. Recruitment relied on open, anonymous, voluntary participation with no follow-up. In total 331 individuals completed and returned surveys (53% of those approached). However, only 222 of these participants completed the six questionnaires of interest (WOMAC, AQoL, SF-36 and the three visual analogue scales) where scores were calculable. Given our objective of validating the AQoL against the other instruments we restricted our analyses to those participants with complete response sets (n = 222). The Lequesne index for hip OA was only completed by participants with hip OA (n = 115). Participants (20%) on the orthopedic waiting list represent the high severity extreme, with their disease clinically judged to require joint replacement surgery. By contrast, the community-based sample (50%) was regarded as having less severe disease. Institutional Ethics Committee approval was given by the Human Research Ethics Committee at the Royal Melbourne Hospital.

### Measures

The AQoL instrument is a generic 12-item utility HRQoL tool incorporating four dimensions: independent living, social relationships, physical senses and psychological well-being [5]. These subscales are weighted between 0.0 (death) and 1.0 (full health). Scores for the four dimensions are combined into an overall utility score extending from minus 0.04 (worst possible HRQoL state) through 0.00 (equivalent to death) to 1.00 (full HRQoL). The time trade-off method was used to determine utility weights in a general population sample. The instrument has been well validated in a range of settings for delivery via self-administration, face-to-face or by proxy and takes approximately 5 minutes to complete [8,10,11].

The WOMAC (Western Ontario McMaster Universities Osteoarthritis Index) is a self-rating instrument designed for patients with lower extremity disease. WOMAC consists of three subscales: pain, stiffness and physical functioning [4]. Subscale scores sum multi-item responses, and a global WOMAC score summates the subscales. Global scores range from 0 (no disease) to 96 (worst disease) and are standardized (0 – 100). The instrument consists of 24 items and takes 5 to 7 minutes to complete. Validation studies have supported the validity, reliability and responsiveness of the WOMAC [3,4,13].

There are two Lequesne's severity indices which were developed for use in patients with (1) hip and (2) knee OA [14]. Each index generates a comparable single summated score which comprises three dimensions: pain/discomfort, maximum distance able to walk and activities of daily living. Scores extend from 00 (no disease) to 24 (worst disease). Categorized scores indicate level of handicap: 1–4 minor, 5–7 moderate, 8–10 severe, 11–13 very severe and > 14 extremely severe. Scores above 10 to 12 indicate surgical treatment is required [14]. Although designed as a clinical assessment tool it has been considered appropriate for self-administration and has been validated and applied in clinical trials [14].

The SF-36 is a widely used generic health status instrument [5,15,16]. It comprises eight multi-item subscales each containing between two and ten items: physical functioning, role physical, bodily pain, general health, vitality, social functioning, role emotional and mental health. Subscales are scored separately, weighted, summed and transformed onto a 0 (poor health) to 100 (excellent health) scale. Two summary composite scores can be constructed: mental component score (MCS) and physical component score (PCS). The SF-36 is designed for self-administration and takes approximately six to nine minutes to complete [16,17].

Three classical visual analogue scales (VAS) covering pain and movement restriction were included. Respondents indicated their experience of pain and restriction over the past week by placing a mark along a horizontal 10 cm line anchored by 0 (no pain) and 100 (worst possible pain). VAS are favored for their simplicity and high correlation with verbal rating scales [18].

### Statistical analyses

Spearman's rank correlation coefficients were used to assess construct validity between the AQoL and WOMAC, SF-36 and VASs. AQoL dimensions intended to measure a similar phenomenon to other scales, were expected to correlate highly. Similarly, AQoL dimensions were expected to have weak correlations with divergent dimensions on other scales. For example, the AQoL's independent living subscale was expected to correlate highly with the WOMAC and SF-36 physical function scores and to exhibit weak correlation with the SF-36 social function. Given the Lequesne and AQoL scales both measure handicap we expected a high correlation between their summary scores [6,14]. Given that the AQoL measures overall Health-related Quality of Life, we expected that the utility score would be moderately correlated with individual elements of OA-related HR-QOL in each of the comparator scales; i.e., health status (SF-36) and arthritis symptoms of pain and limitations (WOMAC, VAS). The classification of the magnitude of correlation coefficients was based on Cohen's rule where < 0.3 is considered a low correlation, 0.3 to 0.6 moderate and > 0.6 high [19].

We assessed the ability of each questionnaire to detect differences between groups through a series of known group validations [20,21]. One set of comparisons was based on self-rated health measured by a five-point scale converted to a dichotomous variable, poor/fair health vs. good/very good/excellent health. The second comparison was between people on the orthopedic waiting list (OWL) with OA and individuals in the community-based group. Known group validation included comparison of AQoL mean scores against the other scales using Student's t tests. We also calculated effect size (ES), relative efficiency (RE) and receiver operator characteristics (ROC) curves to quantify the magnitude of the discriminative performance of the instruments.

Effect size (ES) is the standardized mean difference between groups. We used the pooled standard deviation as the measure of variance. Effect size values of less than 0.2 can be considered small, values of 0.5 as moderate and scores of 0.8 and greater as large [20,22]. Relative efficiency (RE) is the ratio of the squares of the t statistic, where in each case the comparison is against the AQoL score. The RE is commonly used to compare the relative abilities of instruments to detect differences between groups known to be different [20]. Receiver operator characteristics (ROC) curves synthesize information about the sensitivity (proportion of true differences detected by the instrument) and specificity (proportion of individuals truly not different and detected as such) of an instrument against an external 'known difference' criterion, such as categorical differences in severity of disease [20,23]. The power of discrimination can be represented by the area under the ROC curve (AUC), which plots sensitivity against 1 minus specificity. An AUC of 1.0 (100%) represents perfect discriminatory performance and an AUC of 0.5 (50%) implies that an instrument can discriminate between groups no better than would be expected by chance. Using the standard errors for the estimated AUCs we also performed tests to compare the relative performance of the AQoL utility scale against the other instruments. Z statistics were calculated with the critical value set at Z > 1.96, p < 0.05. Given that all instruments were applied to the same data, we adjusted standard errors to account for intra-group correlation before calculating Z statistics [24]. Relative performance was also estimated by the absolute performance gain (APG) of the AQoL against the other instruments [25]. APG analysis compares instrument's AUCs against a nominated reference AUC to evaluate the relative information gain of one instrument over another [25]. In this study we nominated the AQoL as the reference AUC.

This series of tests was repeated in parallel for a subset of the respondents, who had completed the Lequesne index (hip OA only). For this subset, the comparisons were limited to the AQoL, Lequesne's index, and WOMAC summary score.

Liang et al suggest that where a number of tests are conducted to assess instrument performance then a simple method of comparison is to rank order the relative performance of the instruments within each test [23,26]. We present this information for each of the known group analyses. We also compare the AQoL with disease severity by examining the relationship between AQoL and Lequesne instrument scores grouped by previously defined severity of handicap [14].

Statistical analysis was performed using SPSS version 11 software.

## Results

### Participants

Demographic and clinical details of the study participants are presented in Table 1. The majority of respondents (50%) were from the community whilst 20% and 30% came from the Orthopedic waiting list (OWL) and specialist rheumatology clinics respectively. Mean age was 66 (range 42–90) and 67% were female. Approximately three quarters of respondents reported ≥ 2 joint sites affected by OA. A total of 115 reported hip OA and had completed the Lequesne questionnaire that categorized 68% of these individuals as having either extreme or very severe handicap. Of the total sample, 24% reported either 'poor' or 'fair' general health.

**Table 1:** Demographic and clinical details of the study sample

		**Total (N = 222)**		**Rheumatology Clinics** ** (N = 66)**		**Orthopedic Waiting** ** List (N = 45)**		**Community Group** ** (N = 111)**	
		No.	%	No.	%	No.	%	No.	%
									
**Age group (years) **[missing values = 3]	< 55	30	14	11	17	6	14	13	12
	55 to 59	29	13	9	14	3	7	17	15
	60 to 64	33	15	11	17	5	11	17	15
	65 to 69	35	16	8	12	8	18	19	17
	70 to 74	39	18	11	17	7	16	21	19
	75 to 79	32	15	6	9	10	23	16	15
	≥ 80	21	10	9	14	5	11	7	6
									
**Gender**	Female	149	67	44	67	24	53	81	73
	Male	73	33	22	33	21	47	30	27
									
BMI groupings*									
Underweight	< 18.5	0	0	0	0	0	0	0	0
Normal	18.5 to 24.9	64	31	22	37	10	24	32	30
Overweight	25.0 to 29.9	76	37	26	43	18	43	32	30
Obesity I	30 to 34.9	40	19	9	15	11	26	20	19
Obesity II	35.0 to 39.9	19	9	2	3	3	7	14	13
Extreme Obesity [missing values = 14]	≥ 40	9	4	1	2	0	0	8	8
									
**Most common joints affected**	Knee	155	70	44	67	28	62	83	75
	Hip	118	53	25	38	16	36	68	61
	Back	110	50	24	36	16	36	70	63
	Hand/wrist	109	49	25	38	16	36	68	61
	Hip, knee, foot/ankle	179	81	35	53	38	84	101	91
									
**Lequesne scores **grouped for severity of handicap (hip only) [n = 115, missing values = 14]	Extremely severe	53	46	11	46	17	61	25	40
	Very severe	25	22	7	29	5	18	13	21
	Severe	21	18	2	8	6	21	13	21
	Moderate	13	11	2	8	0	0	11	17
	Mild	3	3	2	8	0	0	1	2
									
**Self-rated general health**	Excellent health	15	7	8	12	2	4	5	5
	Very good health	60	27	19	29	10	22	31	28
	Good health	93	42	25	38	20	44	48	43
	Fair health	40	18	10	15	10	22	20	18
	Poor health	14	6	4	6	3	7	7	6

* BMI classifications as per National Heart, Lung, and Blood Institute (USA) Guidelines

The mean (sd) AQoL score was 0.47 (0.22). These data were not significantly skewed and scores did not cluster at scale extremes suggesting floor or ceiling effects. Other summary scales also had normal distributions. The mean scores of the SF-36 PCS and MCS were 30.7 (9.0) and 47.6 (12.2) respectively. WOMAC mean composite score was 45.4 with standard deviation 18.3. The mean Lequesne index score was 12.4 (4.1). The VAS mean scores for average pain, pain at rest and activities of daily living (ADL) were 50.3 (23.6), 32.2 (23) and 43.3 (25.8) respectively.

### Construct validity

Associations between the AQoL and the other instruments are shown in Table 2. It was hypothesised that AQoL Independent living scale would be highly correlated with like scales; this was observed for the SF-36 Physical function scale, WOMAC Physical function and summary score, VAS Restriction in ADL (all R = 0.59) and also the Lequesne scale (R = -0.71). The AQoL Psychological well-being was highly correlated with SF-36 bodily pain (R = 0.60) and WOMAC pain (R = -0.58) and summary score (R = -0.59). The AQoL Physical senses scale is unlike any of the comparators and was consistently weakly associated with all other scales (R < = 0.26).

**Table 2:** Spearman's correlation coefficients between scales

	Independent living	Social relationships	Physical senses	Psychological wellbeing	AQOL Utility
AQoL					
Independent living					
Social relationships	0.48				
Physical senses	0.10	0.20			
Psychological wellbeing	0.46	0.47	0.17		
AQOL Utility	0.78	0.75	0.38	0.77	
SF-36					
Physical Function	0.59	0.34	0.17	0.43	0.59
Role Physical	0.38	0.32	0.05	0.31	0.39
Bodily Pain	0.54	0.43	0.07	0.60	0.63
General Health	0.31	0.31	0.14	0.48	0.45
Vitality	0.46	0.45	0.17	0.57	0.58
Social Function	0.54	0.55	0.21	0.56	0.65
Role Emotional	0.37	0.44	0.10	0.50	0.51
Mental Health	0.37	0.51	0.21	0.55	0.57
PCS	0.47	0.19	0.06	0.31	0.41
MCS	0.39	0.55	0.20	0.61	0.60
WOMAC					
Pain	-0.49	-0.35	-0.03	-0.58	-0.56
Stiffness	-0.43	-0.27	-0.06	-0.51	-0.51
Physical Function	-0.59	-0.37	-0.10	-0.56	-0.63
Total score	-0.59	-0.37	-0.09	-0.59	-0.63
VAS					
Average pain over past week	-0.50	-0.27	-0.07	-0.60	-0.56
Pain while resting over past week	-0.39	-0.25	-0.09	-0.54	-0.49
Restriction in ADL over past week	-0.59	-0.35	-0.10	-0.53	-0.59
					
Lequesne score (hip only)	-0.71	-0.52	-0.26	-0.66	-0.76

Note: Sample size for correlation between scales is n = 222 except for the Lequesne Index as it only relates to OA hip cases (n = 115).

Using Cohen's convention, the magnitude of the correlations can be viewed as; > 0.5 large, 0.5 to 0.3 moderate and < 0.3 weak [18].

**Figure 1:**
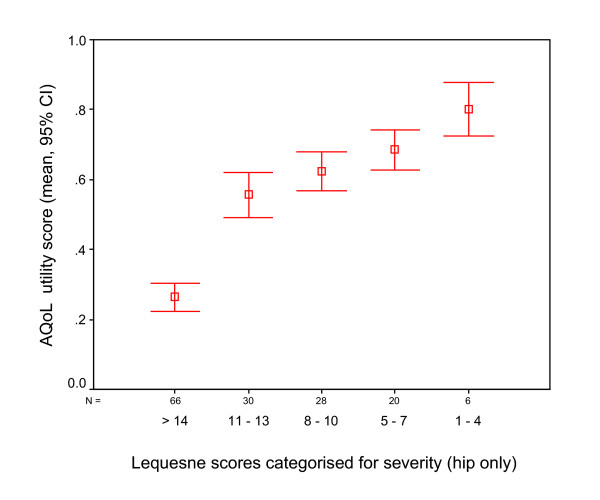
AQoL summary utility score for individuals categorized according to the Lequesne Index of severity (hip), n = 159 (9 missing values).

It was hypothesised that the AQoL utility would exhibit a moderate correlation with comparator scales which were key elements of OA-related HR-QoL (pain and ADL limitations). High to moderate correlations were observed for the WOMAC scales (R < = -0.63), the VAS scales (R > -0.56) and a very high correlation with the Lequesne index (R = -0.76). Grouping of the Lequesne index into severity categories and plotting this against AQoL utility illustrates this relationship (Figure 1).

### Discriminatory Validation

#### Orthopedic Waiting List (OWL) v Community group comparisons

Known-group comparisons of the OWL versus the community-based group are shown in Table 3. The AQoL performed relatively well on the tests of discriminative ability, as did the WOMAC and VASs. The SF-36 performed least well across all tests. Rank ordering of the performance is displayed in Table 4. Effect sizes were largest for the WOMAC physical function (ES, -0.82), its summary score (ES, -0.79) and VAS pain score (ES, 0.74). The AQoL showed moderate effect sizes for its summary score (ES, 0.66) but the Psychological wellbeing scale exhibited the largest effect size (ES, 0.88).

**Table 3:** Ability of the instruments to discriminate between OWL patients and community group

	Waiting List v Community				Comparison of Means				ROC			Absolute Performance Gain			
Instruments	Community (n = 111)		OWL (n = 45)		Effect size #	SD.	*t*	RE	Area Under Curve	Asymptotic 95% CI		*Z*	APG %	95% CI	
	M	SD.	M	SD						Lower	Upper			Lower	Upper
**AQOL**															
**Utility Score**	**0.50**	**0.19**	**0.37**	**0.25**	**0.66**	**0.21**	3.7**	**1.00**	**0.67	**0.57**	**0.77**	**reference**			
Independent living	0.80	0.18	0.69	0.22	**0.58**	0.19	3.3**	0.79	*0.66	0.57	0.75	0.10	1.9	-16.6	20.5
Social relationships	0.84	0.15	0.76	0.26	0.24	0.19	2.4*	0.41	0.56	0.45	0.67	1.08	21.5	1.9	41.1
Physical senses	0.90	0.09	0.92	0.07	-0.26	0.08	-1.4	0.16	0.43	0.33	0.53	**^1.97**	**48.5**	24.4	72.7
Psychological wellbeing	0.81	0.11	0.67	0.23	**0.88**	0.15	4.9**	**1.78**	*0.65	0.54	0.76	0.22	4.4	-15.2	23.9
**SF-36**															
Physical function	40.8	21.0	29.8	24.9	0.50	22.2	2.8*	0.57	**0.67	0.57	0.77	-0.02	0.5	-22.7	23.8
Role physical	26.7	37.1	10.6	22.9	0.48	33.7	2.7*	0.53	*0.61	0.52	0.70	0.47	11.6	-12.3	35.5
Bodily pain	42.7	18.8	33.9	17.2	0.48	18.3	2.7*	0.53	*0.63	0.53	0.72	0.43	8.8	-11.1	28.7
General health	56.6	22.2	51.7	20.8	0.22	21.8	1.3	0.12	0.56	0.47	0.66	0.86	21.1	-3.0	45.2
Vitality	44.7	19.5	43.3	22.0	0.07	20.2	0.4	0.01	0.52	0.42	0.63	1.22	29.4	5.8	53.1
Social function	68.4	24.8	56.4	28.7	0.46	26.0	2.6*	0.49	*0.62	0.52	0.72	0.46	9.8	-11.2	30.9
Role emotional	55.1	44.6	43.0	45.9	0.27	45.0	1.5	0.17	0.57	0.47	0.67	0.81	19.9	-4.2	44.0
Mental health	68.3	17.9	61.3	22.3	0.36	19.2	2.1*	0.30	0.59	0.48	0.69	0.69	16.5	-6.9	39.8
PCS	31.8	9.3	27.8	8.1	0.44	9.0	2.5*	0.46	*0.64	0.55	0.74	0.20	5.0	-19.5	29.4
MCS	47.8	11.6	44.9	13.4	0.24	12.1	1.4	0.13	0.56	0.46	0.66	0.91	21.4	-1.6	44.3
**WOMAC**															
Pain	41.6	20.7	54.3	21.9	-0.60	21.0	-3.4**	0.84	*0.66	0.57	0.76	0.08	1.7	-20.3	0.2
Stiffness	50.0	18.5	59.4	18.9	-0.51	18.6	-2.9*	0.60	*0.63	0.53	0.72	0.37	8.3	-13.5	0.3
Physical function	42.5	19.4	58.5	19.3	**-0.82**	19.4	-4.7**	**1.57**	**0.71	0.62	0.80	-0.38	8.1	-12.8	0.3
Total score	42.9	18.4	57.7	19.0	**-0.79**	18.6	-4.5**	**1.46**	**0.70	0.61	0.79	-0.28	6.0	-14.7	0.3
**VAS **(over past week)															
Average pain	-43.7	23.8	-60.7	21.6	**0.74**	23.2	4.2**	**1.26**	**0.70	0.61	0.79	-0.28	6.0	-0.2	0.3
Pain at rest	-26.9	22.2	-41.8	22.6	0.67	22.3	3.8**	**1.03**	**0.70	0.61	0.78	-0.22	5.0	-0.2	0.3
ADL Restriction	-37.8	26.0	-51.2	23.9	0.53	25.4	3.0*	0.64	*0.65	0.56	0.74	0.18	3.9	-0.2	0.3

^# ^Note: For the effect size calculations the Community group was nominated as the 'control group'. * p < 0.05, ** p < 0.001, ^ p > 0.05.

**Table 4:** Rank ordering of instrument performance by various discriminative tests for the known group comparisons

		Effect Size	RE	ROC (AUC)
Self Rated Health	1	AQoL (-1.00)	AQoL (1.00)	AQoL (0.76)
	2	WOMAC (0.92)	WOMAC (0.84)	WOMAC (0.75)
	3	PCS (-0.84)	PCS (0.71)	PCS (0.67)
	4	VAS pain (0.79)	VAS pain (0.63)	VAS pain (0.65)
OWL v Community	1	WOMAC (-0.79)	WOMAC (1.57)	WOMAC (0.70)
group	2	VAS pain (0.74)	VAS pain (1.03)	VAS pain (0.70)
	3	AQoL (0.66)	AQoL (1.00)	AQoL (0.67)
	4	PCS (0.44)	PCS (0.46)	PCS (0.64)
	5	MCS (0.24)	MCS (0.13)	MCS (0.56)

Note: Comparison groups were Self-rated good vs. poor health and Orthopedic waiting list (OWL) vs. Community based group.

The Relative Efficiency (RE) statistic provided an additional comparison of the ability of each scale to detect differences between groups. The RE of the AQoL utility was referenced to 1.0 which revealed that this scale performed better than all SF-36 scales (RE, 0.01–0.57), the WOMAC pain (RE, 0.84) and stiffness (RE, 0.60) subscales and the VAS ADL scale (RE, 0.64). WOMAC summary (RE, 1.46) and VAS pain scale (RE, 1.26) outperformed the other instruments on this measure.

**Figure 2:**
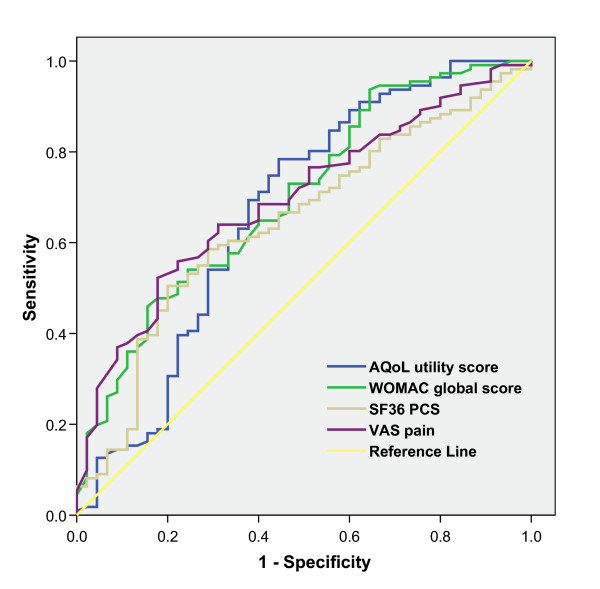
Receiver Operator Characteristic (ROC) curves for performance of the AQoL, WOMAC, SF-36 physical component summary (PCS) score and VAS in discriminating between individuals on the waiting list vs Community.

For the ROC analyses, WOMAC physical function (AUC, 0.71), summary (AUC, 0.70) and VAS pain (0.70) showed the best discrimination. The AQoL utility (AUC, 0.67) also showed substantial discriminatory ability. Of the SF-36 dimensions, Physical function (AUC, 0.67) performed best. A number of subscales and summary scale scores performed no better than chance with AUC values close to 0.50. Figure 2 shows the ROC curves for the AQoL and WOMAC summary scores and the two SF-36 composite measures. Z statistic calculations show that the AUC for the AQoL utility (reference value) was not significantly different from the AUC of other instruments. The sole exception was the AQoL dimension physical senses with Z statistic, 1.96 (Z > 1.96, p < 0.05) performing less well.

Absolute performance gain analyses showed that AQoL utility (reference value) tended to have a positive information gain against other instruments and subscales. Exceptions ranged from SF-36 physical function (0.5%) to WOMAC physical function (8%) which tended to outperform the AQoL utility. Analyses comparing Lequesne's index with the other instruments in the hip disease sub-sample indicated comparable performance with any differences in efficiency not statistically significant (results not shown).

### Self-rated General Health group comparisons

Known-groups comparisons for good health versus poor health are shown in Table 5. The general health and MCS dimensions of the SF-36 were excluded from these analyses, as self-rated general health is a function of the scales. The AQoL performed relatively well on the tests of discriminative ability, as did the WOMAC and VAS. Rank ordering of the instruments is displayed in Table 4. Effect sizes are largest for the AQoL utility (ES, -1.00), WOMAC summary (ES, 0.92) and the SF-36 subscales bodily pain (ES, -1.10) and vitality (ES, -1.12). All other instrument subscale and summary scores show moderate to large effect sizes (ES, 0.53–0.98) with the exception of the AQoL physical senses dimension (ES, -0.23). AQoL utility (RE referenced to 1.0) performed better than WOMAC (RE, 0.65–0.84), VAS (RE, 0.36–0.49) and SF-36 scales (RE, 0.44–0.89) other than SF-36 bodily pain (RE, 1.21) and vitality (RE, 1.26).

**Table 5:** Ability of the Instruments to discriminate between individuals on the basis of self-rated health

	Self-rated General Health				Comparison of Means				ROC			Absolute Performance Gain			
Instruments	Poor health (n = 54)		Good health (n = 168)		Effect size #	SD.	*t*	RE	Area Under Curve	Asymptotic 95% CI		*Z*	APG %	95% CI	
	M	SD.	M	SD.						Lower	Upper			Lower	Upper
**AQOL **															
Utility Score	0.32	0.20	0.52	0.21	**-1.00**	0.20	-6.4	**1.00**	**0.76	0.69	0.83	Reference			
Independent living	0.67	0.22	0.80	0.19	-0.69	0.20	-4.4	0.48	**0.68	0.60	0.77	1.05	15.8	1.1	30.4
Social relationships	0.76	0.21	0.85	0.17	-0.53	0.18	-3.4	0.29	**0.67	0.59	0.75	1.14	17.8	2.4	33.1
Physical senses	0.89	0.10	0.91	0.09	-0.23	0.09	-1.4	0.05	0.56	0.47	0.64	^2.06	40.6	21.3	59.9
Psychological wellbeing	0.66	0.17	0.80	0.13	-0.98	0.14	-6.3	0.97	**0.79	0.72	0.86	-0.38	5.7	-9.2	20.7
**SF-36 **															
Physical function	26.6	17.7	40.8	22.4	-0.66	21.4	-4.3	0.44	**0.69	0.62	0.77	0.79	14.0	-3.4	31.3
Role physical	5.6	15.1	31.1	38.8	-0.74	34.6	-4.7	0.55	**0.69	0.61	0.76	0.79	15.1	-3.7	34.0
Bodily pain	24.8	14.0	43.7	18.1	**-1.10**	17.2	-7.0	**1.21**	**0.80	0.73	0.87	-0.46	7.4	-8.4	23.3
Vitality	28.8	18.0	50.3	19.5	**-1.12**	19.2	-7.2	**1.26**	**0.78	0.72	0.85	-0.25	4.2	-12.5	21.0
Social function	51.9	21.5	69.8	27.1	-0.69	25.8	-4.4	0.48	**0.70	0.63	0.78	0.71	11.4	-4.4	27.2
Role emotional	24.7	39.0	64.0	42.7	-0.94	41.8	-6.0	0.89	**0.73	0.66	0.81	0.28	5.4	-13.2	23.9
Mental health	54.7	17.4	70.6	18.7	-0.86	18.4	-5.5	0.75	**0.74	0.67	0.81	0.25	4.3	-12.8	21.4
PCS	25.3	5.6	32.4	9.2	-0.84	8.5	-5.4	0.71	**0.74	0.67	0.81	0.26	4.9	-1.4	23.4
**WOMAC **															
Pain	57.6	17.9	41.8	20.2	0.81	19.7	5.2	0.65	**0.73	0.65	0.80	0.41	7.2	-10.1	24.5
Stiffness	64.8	18.0	49.0	18.8	0.85	18.6	5.4	0.72	**0.72	0.65	0.80	0.41	7.6	-10.4	25.6
Physical function	59.6	15.5	43.1	19.7	0.88	18.8	5.6	0.77	**0.74	0.67	0.81	0.23	3.9	-12.5	20.4
WOMAC score	59.6	15.0	43.3	18.5	0.92	17.6	5.9	0.84	**0.75	0.68	0.82	0.13	2.1	-14.3	18.5
**VAS **(over past week)															
Average pain	-62.3	21.0	-46.4	23.2	-0.70	22.7	-4.5	0.49	**0.69	0.61	0.77	0.75	13.6	-4.3	31.4
Pain at rest	-45.3	21.7	-28.0	21.8	-0.79	21.8	-5.1	0.63	**0.72	0.65	0.80	0.43	7.8	-10.0	25.7
ADL Restriction	-54.7	23.5	-39.6	25.5	-0.60	25.1	-3.8	0.36	**0.66	0.59	0.74	1.11	19.4	2.3	36.6

N = 222, * All significant < 0.001 except Physical senses, p = 0.15.

^#^Note: For the effect size calculations the poor health group was nominated as the 'control group'.

**Figure 3:**
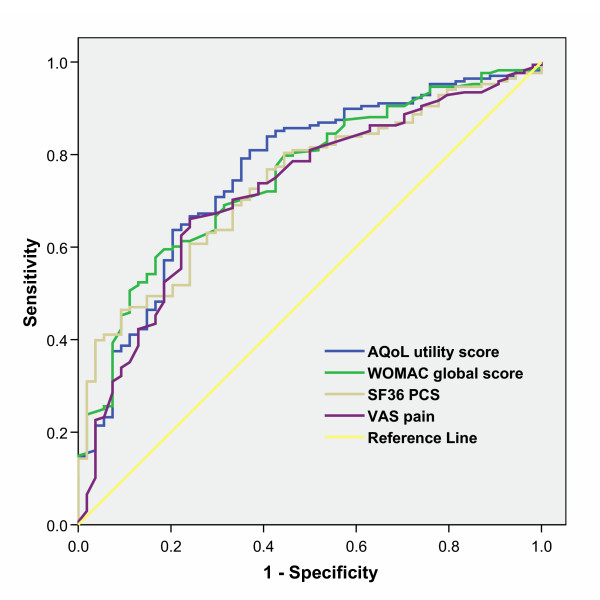
Receiver Operator Characteristic (ROC) curves for performance of the AQoL, WOMAC, SF36 physical component summary (PCS) score and VAS in discriminating between individuals with self rated poor vs good health.

All instruments and dimensions performed similarly in the ROC analyses with AUC ranging from 0.66 to 0.80. The AQoL physical senses (AUC 0.56, p = 0.19) performed no better than chance. The ROC curves for the AQoL and WOMAC summary and the SF-36 composite measures are shown in Figure 3. Z statistic calculations show that the AUC for the AQoL utility (reference value) was not significantly different from the AUC of other instruments or dimensions. The only exception was the weakly discriminating AQoL physical senses scale (Z statistic 2.06; Z > 1.96, p < 0.05). Absolute performance gain analyses show the AQoL utility (reference value) had a positive information gain against most other instruments. Analyses comparing Lequesne's index with the other instruments in the hip disease sub-sample indicated comparable performance with any differences in discrimination and efficiency not statistically significant (results not shown).

## Discussion

This study has provided strong evidence that the AQoL has strong construct validity and strong discriminative validity for use in people with OA. This finding, in conjunction with prior studies indicating the AQoL's responsiveness, suggests the AQoL is a promising tool for OA clinical and epidemiological research. Further, the AQoL is a parsimonious 12-item instrument returning information on four domains and providing a single utility score. When compared with commonly used disease specific (WOMAC & Lequesne) and generic (SF-36) measures the AQoL performs as well or better with high discriminatory validity. The AQoL was also found to have strong convergent and divergent validity supporting its overall construct validity [18]. Furthermore, assuming that the Lequesne algorithm represents severity of handicap due to OA, the high correlation between it and the AQoL utility supports criterion validity of the AQoL in this disease group [2].

The principal aspects of handicap for people with OA results through pain, limitations and the impact that these have on peoples' overall life, including how primary OA symptoms pervade social and psychological well-being [27]. The AQoL is designed to capture these through preferences in a weighted multi-attribute utility measure expressed on a life-death scale. In this way, the AQoL provides a single score that represents the disutility ('undesirableness') of their overall health status.

As there is no consensus on a definitive discriminative test, we applied a range of measures to examine performance of each questionnaire [28]. Rank ordering the instruments within these tests shows the AQoL performs well [26]. Consistent with other studies we also explored ROC and APG analyses to empirically test instruments' relative efficiency and performance [24, 25, 29, 30]. Although the results comparing the known groups based on clinical difference (OWL vs community group) appear to suggest the disease specific outcomes perform better, our empirical analyses show that the difference is not statistically significant.

There has been some debate about the relative merits of using generic versus disease specific measures across settings and disease groups [2,18,23,28]. Disease specific outcome measures are often favored in clinical trials where there is a need for a sensitive measure of small but important clinical changes and a need to rapidly categorize patients [18]. This is because generic instruments are viewed as relatively insensitive to these small 'clinically important' changes and cover irrelevant domains. Other cited advantages of disease specific outcome measures include suitability for self-administration, brevity, simplicity of administration and calculation of summary scores and low cost [18]. However, it is recognized that generic measures facilitate comparison between studies, as they are broadly applicable across a range of severity and type of diseases, populations and different interventions [18]. It is also postulated that the breadth of dimensions explored using generic HRQoL questionnaires can capture the full effects, including unanticipated impacts, of an intervention [2]. A way of resolving the issue of whether generic or disease specific questionnaires should be administered is through combined use of generic and disease specific outcome measures in clinical trials [2,18,28]. Others reject this proposal arguing overlapping content and excessive questionnaire items impose an unacceptable burden on respondents reducing compliance [20]. This suggests that investigators should use the most valid and parsimonious instruments available that are able to detect the hypothesized outcomes. Also where investigators require evidence of the overall and comparative value of an intervention, use of a generic utility instrument is recommended [20]. Given that utility instruments reflect both patients' health status and valuation of health state [2] their scores can be incorporated into cost utility studies facilitating comparative economic appraisals of interventions.

## Conclusion

The AQoL utility instrument exhibits comparable efficiency and performance relative to more commonly used health outcome measures in OA studies, i.e., WOMAC and SF-36. In addition the AQoL has only 12 items compared with the 24 or 36 items of the WOMAC and SF-36 respectively. As a generic index the AQoL facilitates comparisons across groups and also provides a utility score suitable for economic analyses. For these reasons, and prior strong evidence of its validity in other settings including responsiveness [7-9] the AQoL appears to be a suitable adjunct to disease specific questionnaires in clinical trials and epidemiological studies of people with OA.

## Authors' contributions

RO and LS, contributed to the data collection, KW and RO undertook the analysis, RB, KW and RO contributed to the drafting on the manuscript.
